# Computed tomographic appearance of circumcaval and circumuterine ureter in a cat

**DOI:** 10.1002/vms3.273

**Published:** 2020-04-26

**Authors:** Jitrapun Jirasakul, Ninlawan Thammasiri, Damri Darawiroj, Nan Choisunirachon, Chutimon Thanaboonnipat

**Affiliations:** ^1^ Department of Surgery Faculty of Veterinary Science Chulalongkorn University Bangkok Thailand; ^2^ Department of Anatomy Faculty of Veterinary Science Chulalongkorn University Bangkok Thailand

**Keywords:** cat, circumcaval, circumuterine, CT, ureter

## Abstract

**Background:**

Circumcaval ureter is a rare congenital anomaly resulting from the persistence of embryonic venous system. This anomaly is characterized by running of affected ureter around caudal vena cava (CVC).

**Case presentation:**

In this report, circumcaval ureter was found in a 2‐year‐old female cat attending as normal sample group in another research. This cat passed all health checkup protocols before computed tomography (CT) was performed.

**Conclusion:**

The contrast‐enhanced computed tomographic (cCT) images clearly revealed the dislocation of the right ureter that course around CVC and uterine body. This is the first report of feline circumcaval concurrent with circumuterine ureter detected by cCT.

## Graphical abstract

In this report, we have found the circumcaval ureter in a 2‐year‐old female cat attending as normal sample group in another research. This cat passed all health checkup protocols before computed tomography (CT) was performed. The contrast‐enhanced computed tomographic (cCT) images clearly revealed the dislocation of the right ureter that course around CVC and uterine body. This is the first report of feline circumcaval concurrent with circumuterine ureter detected by cCT.

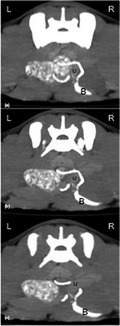

## 1 INTRODUCTION

Circumcaval ureter is a rare anomaly of the embryonic venous system. Generally, there are three embryologic venous systems composing of caudal/posterior cardinal, subcardinal and supracardinal venous systems, which form as caudal vena cava (CVC). In the case of persistence of right caudal cardinal vein, CVC will be ventrally displaced and ureter will be medially run behind the CVC, circumscribed to its normal position (Bass, Redwine, Kramer, Huynh, & Harris, 2000; Hyseni et al., 2013; Ichikawa et al., 2014; Minniti, Visentini, & Procacci, 2002; Nagashima et al., 2006; Ratkal, Jadhav, & Naique Dessai, 2016; Shin, Lee, Park, Park, & Kim, 2014; Steinhaus et al., 2015; Yarmohammadi, Mohamadzadeh Rezaei, Feizzadeh, & Ahmadnia, 2006). This condition could be incidentally found without any signs of urinary tract abnormality (de Arruda et al., 2018). However, ureteral obstruction can be found with signs of flank pain and hydronephrosis (Bhattacharjee, Sanga, Gupta, & George, 2016; Kajal, Rattan, Sangwan, & Bhutani, 2016).

In human medicine, the prevalence of circumcaval ureter was reported as 0.13% (Hostiuc, Rusu, Negoi, Grigorui, & Hostiuc, 2019). Comparing to companion animals, circumcaval ureter has been reported in a dog (Doust, Clarke, Hammand, Paterson, & King, 2006). However, this abnormality has been more frequently reported in cats (Abidu‐Figueiredo et al., 2019; Bélanger, Shmon, Gilbert, & Linn, 2014; Pey, Marcon, Drigo, Specchi, & Bertolini, 2015; Steinhaus et al., 2015). In addition, several reports showed that it could be concurrently occurred with other congenital abnormalities; for example: intrahepatic portosystemic shunt (Doust et al., 2006), double right renal vein (Abidu‐Figueiredo et al., 2019), contralateral renal agenesis (Cardoza, Shambhulinga, & Rajeevan, 2016) and cryptorchidism (Fernando, Jayarajah, Arulanantham, Goonewardena, & Wijewardena, 2018). However, there is no report of feline circumcaval concurrent with circumuterine ureter. Therefore, the aim of this report was to reveal an incidental, contrast‐enhanced computed tomographic (cCT) case of circumcaval concurrented with circumuterine ureter in a cat.

## 2 CASE REPORT

A 2‐year‐old, 4.4 kg, intact female Persian cat was presented at the Small Animal Teaching Hospital, Faculty of Veterinary Science, Chulalongkorn University as the normal volunteer for another research. All processes of the study were conducted under the approval by The Institution Animal Care and Use Committee of Faculty of Veterinary Science, Chulalongkorn University (CU‐IACUC), approval number: 1631073. After history taking, physical examination, routine laboratory tests and the test for feline leukaemia virus (FeLV) and feline immunodeficiency virus (FIV) (Witness^®^ Zoetis) were done and normal ranges of former tests were achieved, the cat was subjected to perform abdominal radiography, ultrasonography and computed tomography (CT), using the previous study protocol (Darawiroj & Choisunirachon, 2019).

The abdominal radiograph includes lateral and ventrodorsal projections was performed using the digital radiograph (Brivo DR‐F, GE, USA; 65 kVp and 10 mAs) without sedation or anaesthesia. In brief, the cat was pre‐medicated with acepromazine maleate (0.03 mg/kg, Combistress^®^ Belgium) and tramadol hydrochloride (2 mg/kg, Tramache^®^). Subsequently, the anaesthetic induction was performed using propofol (2–4 mg/kg, Lipuro^®^) prior endotracheal tabulation. The anaesthesia was maintained by isoflurane (2%–5%, AERRANE^®^). CT was conducted using a 64‐slice, CT scanner (Optima CT660) in prone position with the head point into the gantry and the field of view was set to be covered the whole abdomen. After the pre‐contrast CT (pcCT) images of abdomen were obtained, iohexol contrast medium (600 mgI/kg, Omnipaque 300^®^) was intravenously administrated. Contrast‐enhanced CT images (cCT) were then performed using a low‐pass filter, 1.25‐mm slice thickness, collimator pitch at 0.531, matrix of 512 × 512, peak kilovoltage of 120 kVp and automated amperage.

All imaging data were collected as the Digital and Communication in Medicine (DICOM) files and reanalysed by DICOM viewer software (OsiriX^®^). The cCT was displayed using a soft tissue window at 350 Hounsfield Unit (HU) of window width (WW) and 40 HU of window level (WL). After the abnormality of ureter was detected, maximum intensity projection (MIP) was applied to clearly unveil morphology and location of ureter.

## 3 RESULTS

**FIGURE 1:**
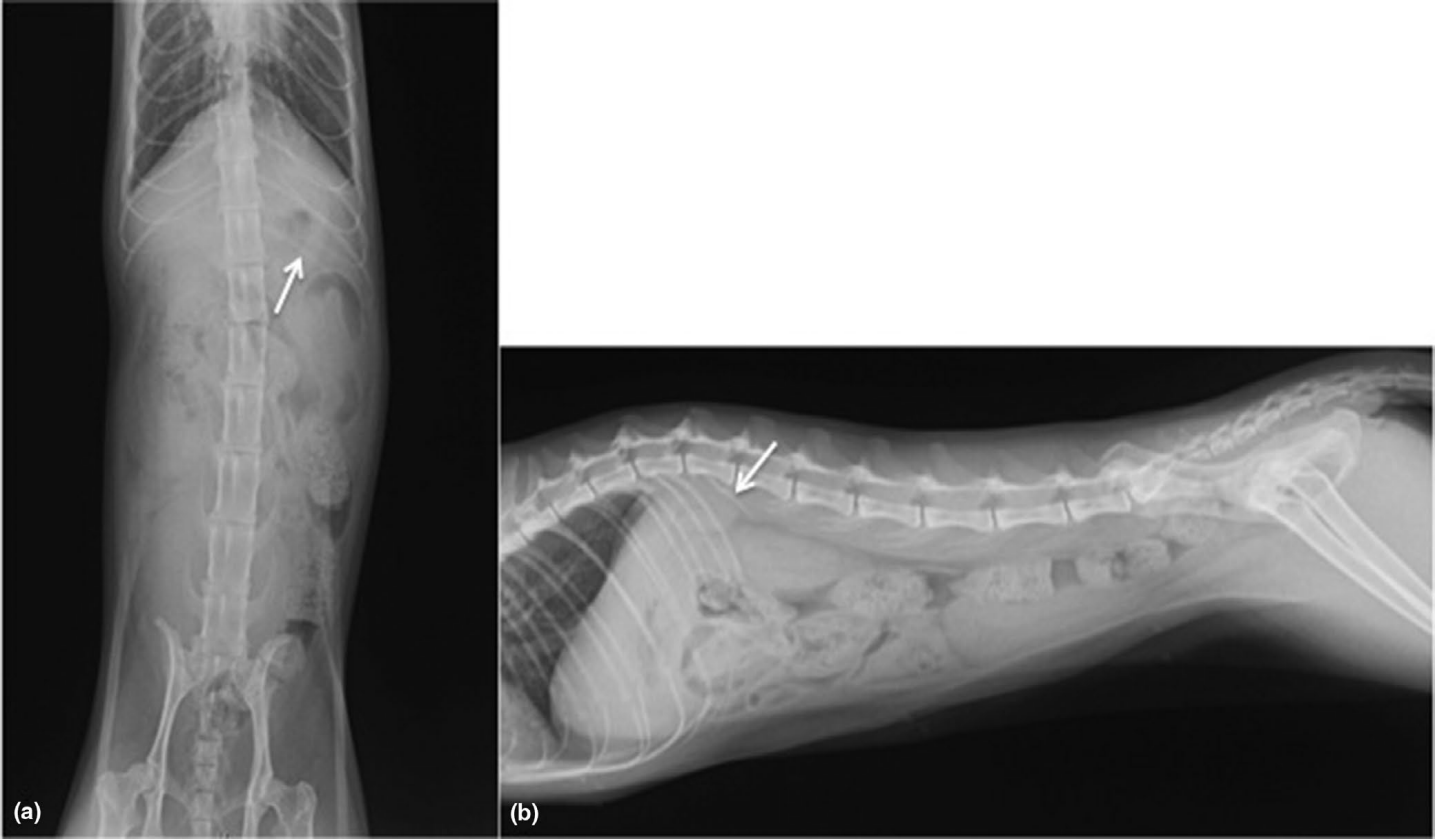
Abdominal radiographs on ventrodorsal (a) and right lateral projection (b) revealed the transitional vertebrae of the first lumber vertebra with a left rib (arrow)

Plain abdominal radiographs showed unremarkable abnormalities excepted for a transitional first lumbar vertebra (L1) with a left rib (Figure 1). The left kidney was located more slightly cranial than the right kidney (Figure 1). Renal length of the left and the right kidneys were 3.63 and 3.73 cm, respectively. Ultrasonographically, unremarkable intra‐abdominal organs were noted excepted for a trace amount of cystic urine sediment.

**FIGURE 2:**
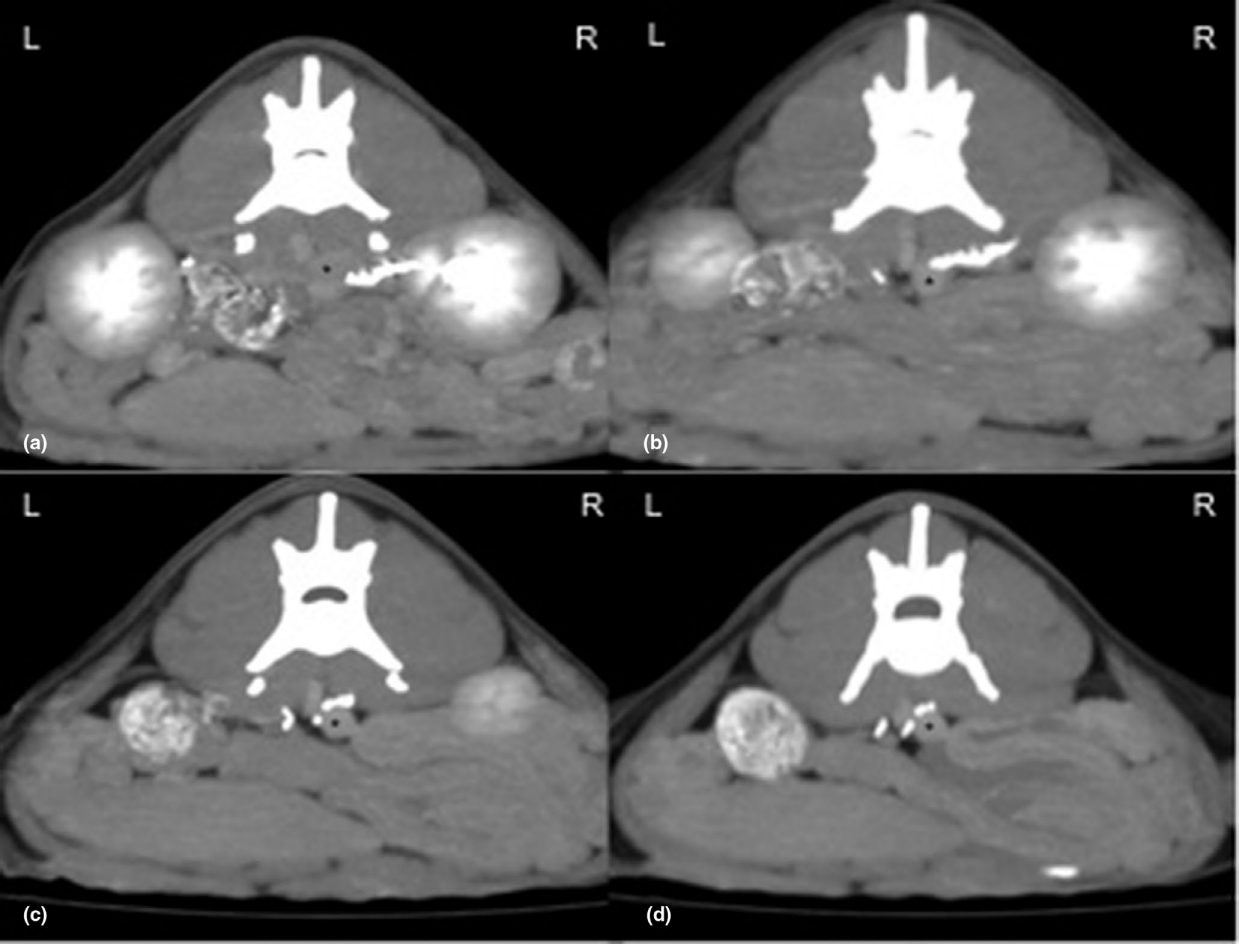
Axial plane of delayed contrast enhanced computed tomographic images (a–d) of the mid abdomen indicated that the right circumvaval ureter was ran from the right retroperitoneum to the left side which dorsally to the caudal vena cava (asterisk)

**FIGURE 3:**
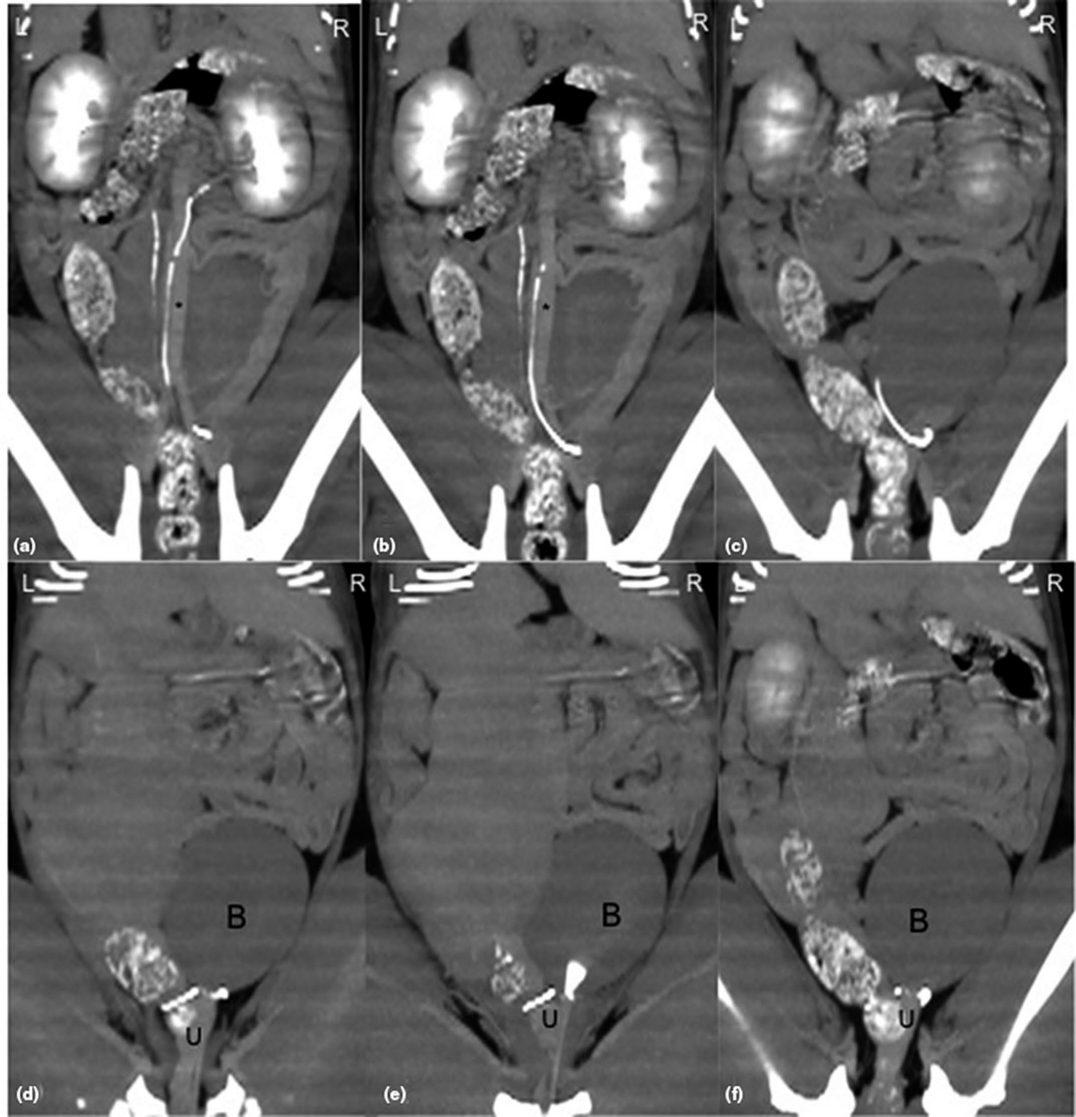
Dorsal plane of delayed contrast enhanced computed tomographic images (a–c) of the mid abdomen showed that the right circumcaval ureter was firstly circumscribed the caudal vena cava (asterisk) as the inverted‐J shape prior secondly turned around the uterine body (U) to the normal position on the right side (d–f); B, bladder

**FIGURE 4:**
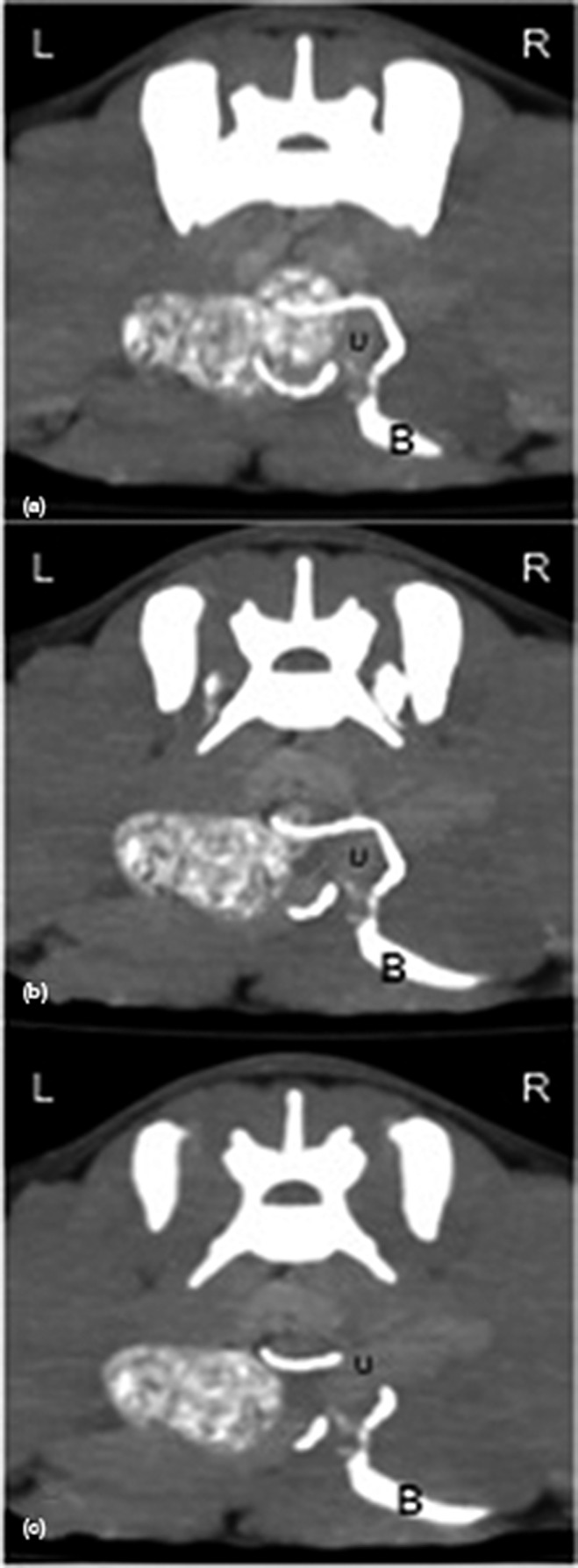
Axial plane of delayed contrast enhanced computed tomographic images (a–c) of the caudal abdomen indicated that the right circumcaval ureter lied around the uterine body (U) prior entering the right vesicoureteral junction of the bladder (B)

After CT scannning, both pcCT and cCT, the pcCT showed unremarkable changes of abnormal organs, cCT images showed the normal shape and position of the left ureter, whereas the right ureter which was more distinct due to the delayed excretion of contrast medium initially ran from the right retroperitoneal area to the left side, dorsal to CVC at the level of the 5th lumbar vertebra (L5) (Figures 2 and 3). Subsequently, it continued downward to midline and then turned right laterally to the normal alignment by ventrally circumscribing the uterus at the level of the first sacral vertebra (Figure 4). Finally, the ureter entered through the right vesicoureteral junction of the bladder. In addition, no evidence of hydronephrosis or obstructive signs of urinary bladder were detected.

## 4 DISCUSSION

CVC is formed from three embryologic venous systems, which are caudal or posterior cardinal, subcardinal and supracardinal veins. These systems undergo continuous appearance, regression and anastomosis during foetal development (Bass et al., 2000; Casteleyn, Cornillie, Cruchten, & Ginneken, 2015; Chou, Yang, Hong, & Wu, 2006; Ichikawa et al., 2014; Minniti et al., 2002; Nagashima et al., 2006; Shin et al., 2014; Srivastava, Singh, Suri, Vijjan, & Dubey, 2005; Steinhaus et al., 2015). If there is an incomplete development, these veins will be remained and caused various types of congenital anomalies. It may result in at least 14 anatomic anomalies in humans such as duplication of the CVC, left‐side CVC and circumaortic left renal vein (Srivastava et al., 2005).

The normal CVC is composed of four segments: hepatic, suprarenal, renal and infra‐renal segments. The hepatic segment is derived from the vitelline vein. The suprarenal segment is derived from the right subcardinal vein. The renal segment is derived from the right supra, subcardinal and caudal subcardinal anastomosis. The infrarenal is derived from the right supracardinal vein. The common variations of infrarenal segments are four types; the persistent right supracardinal vein (normal CVC), the persistent right caudal cardinal vein (circumcaval ureter), the persistent left supracardinal vein (left CVC) and the persistent right and left supracardinal veins (double CVC) (Dudekula and Prabhu, 2014; Pey et al., 2015; Shin et al., 2014). Thus, the congenital anomaly found in this cat could be the persistent right caudal cardinal vein during embryonic development. According to Bélanger and coworkers in 2014, the circumcaval ureter might be a common vascular anomaly in cat because the prevalence in cat was more than one third.

In human, two types of circumcaval ureter were reported. Type 1 is a low loop resembled the reverse J, S or hook shape of ureter which is the most common of circumcaval ureter in human (Basok, Yildirim, & Tokuc, 2008; Pey et al., 2015; Steinhaus et al., 2015; Uthappa, Anthony, & Allen, 2002). Moreover, half population of type 1 abnormality may develop hydronephrosis (Ratkal et al., 2016; Steinhaus et al., 2015; Uthappa et al., 2002). Type 2 is a high loop, sickle‐shaped ureter that crosses the CVC at the ureteropelvic junction (UPJ). This type hardly demonstrated sign of urinary tract obstruction (Basok et al., 2008; Casteleyn et al., 2015; Pey et al., 2015; Steinhaus et al., 2015; Uthappa et al., 2002).

The right ureter of this cat was S‐shaped and crossed the CVC at the level of L5, thus this abnormality was type 1 pattern. This location or the area of the fourth‐lumbar vertebra (L4) to L5 was the common location of the circumcaval ureter (Pey et al., 2015). Additionally, before entering the vesicoureteral junction, the ureter was turning to its normal position by circumscribing the uterus. This may cause ureteral obstruction and hydronephrosis during active gonad especially during oestrus cycle or pregnancy. In case of pregnancy without notice of this abnormality, uterine obstruction may cause more injuries. Moreover neutering by ovariohysterectomy (OVH) in circumuterine ureter, unintentionally trauma by suturing or cutting an affected ureter can be harmful. Therefore, to perform OVH or other urethral surgeries such as traumatic causes, the surgeon should particularly concern. Surprisingly, this cat was alert and had no sign of urinary tract obstruction.

It was probably that the cat was in the early age. It has been reported that circumcaval ureter induced hydronephrosis would be more prone in the middle age (Bélanger et al., 2014; Hyseni et al., 2013; Ratkal et al., 2016; Steinhaus et al., 2015). Furthermore, one study revealed that mean right kidney length of the cat with right circumcaval ureter was significantly longer than the left (Bélanger et al., 2014; Hyseni et al., 2013; Ratkal et al., 2016; Steinhaus et al., 2015). However, the kidney sizes of this cat were comparable. This may be due to no obstruction at the ureter. From the time of CT until this report was written or about two years, the cat was rechecked for the sign of urinary obstruction by laboratory tests and abdominal ultrasonography, unremarkable changes were noted. Nevertheless, the owner was informed to be close monitoring for observing the prospective abnormalities that may be induced by circumcaval and circumuterine such as ureter hydroureter, hydronephrosis or proximal urolith of urinary system; both of renal and ureteral calculi.

Diagnosis method for this anomaly was challenging, because many cats were asymptomatic and/or the affected, non‐obstructive ureter were not easily visualized on routine radiographs or ultrasonograms. Plain radiograph can detect only mineral opacities. Excretory urography on radiographs was also not sufficient; the visualization depended on the severity of lesion, ureteral peristalsis and radiological projection. Ultrasonography was not clearly seen due to its small size and the presence of gas or faecal material. Previous reports suggested that circumcaval ureter might be suspected in the case of proximal ureteral obstruction and presented of a calculus at the site of obstruction (Pey et al., 2015; Steinhaus et al., 2015).

While other imaging procedures cannot clearly identify, cCT scan acted as a superior modality for detecting both of vasculature and urinary system. Although pcCT provided a good anatomical image of kidney and main abdominal vasculature without superimposition, this anomaly could not be easily found without contrast enhancement due to the small structures with the same attenuation of soft tissue. cCT enhanced the visualization of CVC and also the excretion of contrast medium in both ureters. cCT was a common imaging modality to detect circumcaval ureter in human by observing the excretory phase of ureter (Agrawal, Kanojia, & Saxena, 2017). The right ureter of this cat was more obviously detected because the left had normal peristalsis while the right had delayed peristalsis, affected from its deviation crossing CVC.

In addition to cCT, magnetic resonance imaging (MRI) showed an equal effectiveness to detect the lesion without radiation risk (Hyseni et al., 2013; Mathusami & Ramesh, 2013; Ratkal et al., 2016; Uthappa et al., 2002). However, MRI is not widely used in veterinary medicine, especially in Thailand due to the higher cost and longer time of the procedure. Therefore, prospective study using MRI for diagnosis of this anomaly will provide more valuable information for small animal diagnostic imaging. In addition to the right circumcaval and circumuterine ureter, this cat also affected with the presence of a left rib at the L1. Therefore, it would be implied that during the foetal development, this cat might be faultily developed in anatomical structures.

## 5 CONCLUSION

Circumcaval ureter might be a common anomaly in cats, which was hardly detected by routine diagnostic modalities and could cause ureteral obstruction. This anomaly should be suspicious in cats with persistent urinary signs or proximal ureteral obstruction with or without presence of ureteral calculus. To detect circumcaval ureter clearly, cCT was an excellent imaging tool. To the best of our knowledge, this case was the first report of feline circumcaval concurrent with circumuterine ureter.

## CONFLICT OF INTEREST

The authors declare that there is no conflict of interest that could be perceived as prejudicing the impartiality of the research reported in this article.

## AUTHOR CONTRIBUTION

Jitrapun Jirasakul: Project administration; Visualization; Writing‐original draft; Writing‐review & editing. Naparee Srisowanna: Formal analysis; Investigation. Ninlawan Thammasiri: Conceptualization; Data curation; Formal analysis; Investigation; Methodology; Project administration; Writing‐original draft. Damri Darawiroj: Methodology; Project administration; Visualization; Writing‐original draft; Writing‐review & editing. Nan Choisunirachon: Conceptualization; Data curation; Methodology; Software; Supervision; Validation; Visualization; Writing‐review & editing. Chutimon Thanaboonnipat: Conceptualization; Formal analysis; Investigation; Methodology; Validation; Visualization; Writing‐original draft; Writing‐review & editing.
